# Impact of left atrial appendage occlusion device position on potential determinants of device-related thrombus: a patient-specific in silico study

**DOI:** 10.1007/s00392-023-02228-x

**Published:** 2023-06-09

**Authors:** Zhaoyang Zhong, Yiting Gao, Soma Kovács, Vivian Vij, Dominik Nelles, Lukas Spano, Georg Nickenig, Simon Sonntag, Ole De Backer, Lars Søndergaard, Alexander Sedaghat, Petra Mela

**Affiliations:** 1 Chair of Medical Materials and Implants, TUM School of Engineering and Design and Munich Institute of Biomedical Engineering, Technical University of Munich, Garching, Germany; 2 Department of Cardiology, University Hospital Bonn, Bonn, Germany; 3 Virtonomy GmbH, Munich, Germany; 4 Department of Cardiology, Rigshospitalet, Copenhagen University Hospital, Copenhagen, Denmark; 5 RheinAhrCardio, Praxis für Kardiologie, Bad Neuenahr-Ahrweiler, Germany

**Keywords:** Left atrial appendage occlusion, Device-related thrombus, Computational fluid dynamics, Patient-specific modeling

## Abstract

**Background:**

Device-related thrombus (DRT) after left atrial appendage occlusion (LAAO) is potentially linked to adverse events. Although clinical reports suggest an effect of the device type and position on the DRT risk, in-depth studies of its mechanistic basis are needed. This in silico study aimed to assess the impact of the position of non-pacifier (Watchman) and pacifier (Amulet) LAAO devices on surrogate markers of DRT risk.

**Methods:**

The LAAO devices were modeled with precise geometry and virtually implanted in different positions into a patient-specific left atrium. Using computational fluid dynamics, the following values were quantified: residual blood, wall shear stress (WSS) and endothelial cell activation potential (ECAP).

**Results:**

In comparison to an ostium-fitted device position, deep implantation led to more residual blood, lower average WSS and higher ECAP surrounding the device, especially on the device’s atrial surface and the surrounding tissue, suggesting increased risk for potential thrombus. For the non-pacifier device, an off-axis device orientation resulted in even more residual blood, higher ECAP and similar average WSS as compared to an ostium-fitted device position. Overall, the pacifier device showed less residual blood, higher average WSS and lower ECAP, compared to the non-pacifier device.

**Conclusions:**

In this in silico study, both LAAO device type and implant position showed an impact on potential markers of DRT in terms of blood stasis, platelet adhesion and endothelial dysfunction. Our results present a mechanistic basis for clinically observed risk factors of DRT and the proposed in silico model may aid in the optimization of device development and procedural aspects.

**Graphical abstract:**

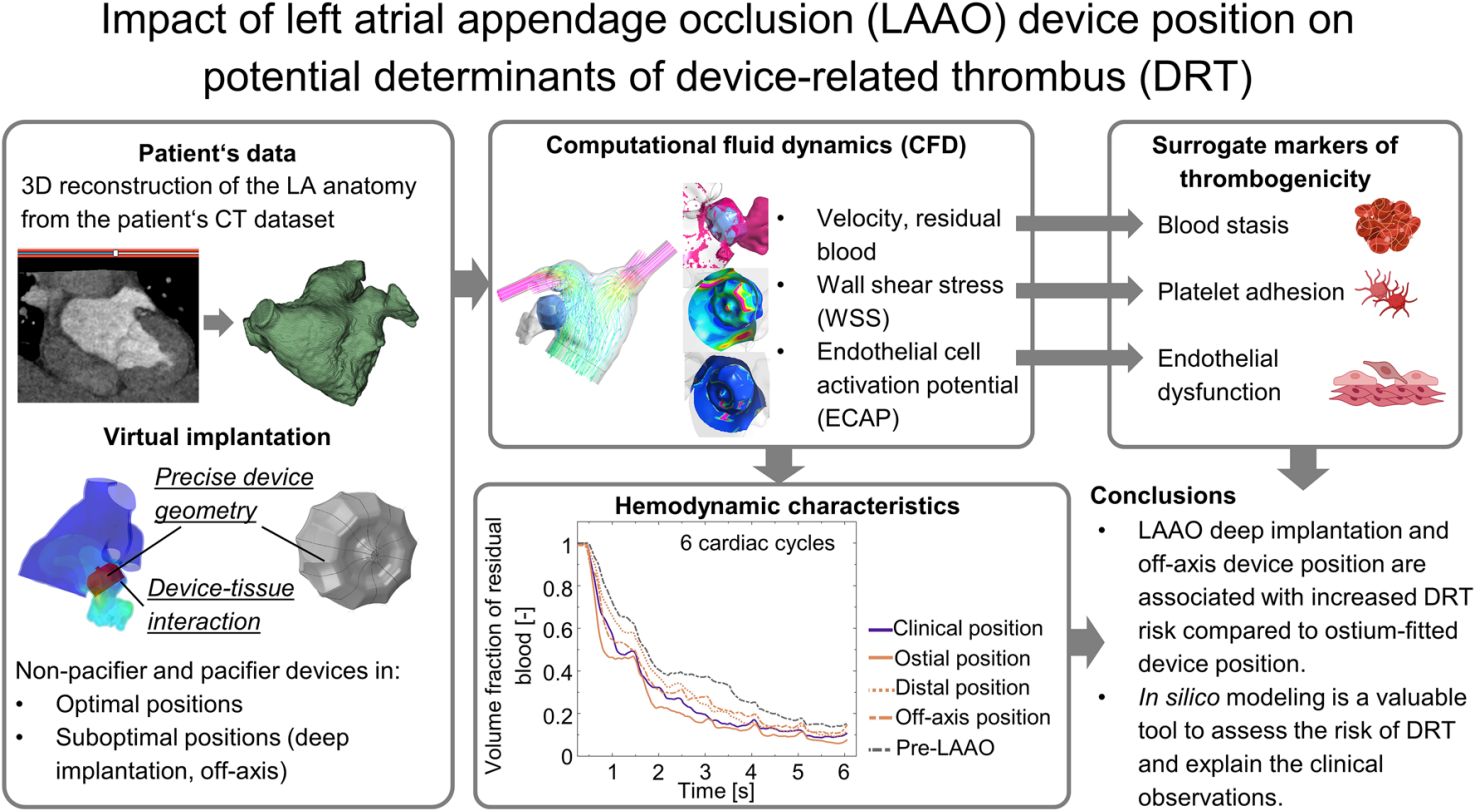

**Supplementary Information:**

The online version contains supplementary material available at 10.1007/s00392-023-02228-x.

## Introduction

Atrial fibrillation (AF) is the most common cardiac arrhythmia and is associated with a risk of embolic stroke. About 90% of all cardiac emboli in AF are found in the left atrial appendage (LAA), mostly owing to the local pro-coagulatory hemodynamic conditions [1, 2]. Oral anticoagulation using direct oral anticoagulants (DOACs) or vitamin K antagonists is the gold standard for preventing embolic stroke in AF [3]. For patients with contraindications to anticoagulation or at excessive risk for bleeding, percutaneous left atrial appendage occlusion (LAAO) has become a valuable alternative, reducing the risk of embolism by physically excluding the LAA from the bloodstream [4].

Recently, the relevance of device-related thrombus (DRT) after LAAO has been highlighted with an incidence ranging from 2 to 16% and the presence of DRT seems to be linked to an increased risk of stroke and adverse outcome [5–7]. It is consensus that DRT is of multifactorial cause including procedural and technical parameters, such as device type and position. Recent clinical observational series have provided evidence on the influence of device position and local hemodynamics on DRT [7–11]. Blood stasis, platelet adhesion, as well as endothelial cell damage and dysfunction are promoted in the case of AF [12, 13], these factors may play an important role in thrombus formation after LAAO.

Despite the clinical observations, the mechanistic details of the impact of local hemodynamics on DRT formation remain poorly understood. To comprehend these effects, in-depth studies of the hemodynamics and the surrogate markers of thrombogenicity, including blood stasis, platelet adhesion and endothelial dysfunction, are needed. Given the manifold anatomic variations of the LAA, the restrictions of clinical hemodynamic measurement and the limited predictability of LAAO device position, comparative clinical assessment of the influence of device position on DRT is cumbersome.

The potential of computational fluid dynamics (CFD) as a tool to assess DRT risk has been shown in recent studies where blood stasis and endothelial dysfunction were considered as markers of DRT [14–19]. Some of these studies highlighted the importance of the LAAO device position [14, 16–19]. So far, an in silico study that includes device position, type and size as DRT risk factors within the same anatomy is lacking. In addition, current in silico models are not able to recapitulate some clinically observed DRT locations, such as the central screw cove and the shoulder of a non-pacifier device [20–22], as well as the threaded insert of a pacifier device [7]. The precise geometry of the LAAO device and its interaction with the tissue upon virtual implantation are likely needed to make an in silico model able to recapitulate the clinically observed DRT locations.

Therefore, in this work we developed an in silico model to realistically assess the impact of the device position on the hemodynamics by including the precise morphology of non-pacifier and pacifier LAAO devices, as well as the device-tissue interaction. In this way, we aim to provide a mechanistic explanation for the clinically observed DRT occurrence and its dependence on the position of the LAAO device.

## Methods

The computational framework of this in silico study began with medical images analysis and 3D anatomic reconstruction. Following this step, the computer-aided design (CAD) model of the Watchman Gen 2.5 (Boston Scientific, Marlborough, MA, USA) and Amplatzer Amulet (Abbott, Abbott Park, IL, USA) LAAO devices were virtually implanted in different positions using finite element method (FEM). CFD simulations were subsequently performed to estimate the hemodynamics and the surrogate markers of thrombogenicity (Table 1) for each device position under AF conditions.

**Table 1:** Surrogate markers of thrombogenicity

Hemodynamic characteristics	Calculated variables	Surrogate marker of thrombogenicity
Flow patterns	Flow velocity *u* Vorticity *ω* = ∇ × *u*	Blood stasis
Blood washout and residence	Ratio of residual blood $\varphi = \frac{{V}_{\text{old blood}}}{{V}_{\text{ROI}}}$ Washout halftime ${t}_{h (\varphi =50\%)}$	Blood stasis
Wall shear stress	WSS ${\tau }_{w}=\mu {(\frac{\partial u}{\partial y})}_{y=0}$	Adhesion and stabilization of transient discoid platelet aggregates in low shear area, thereby thrombus growth
Endothelial cell activation potential	$\text{ECAP}=\frac{\rm{OSI}}{\rm{TAWSS}}$ $\text{OSI}=\frac{1}{2}\left(1-\frac{|\underset{0}{\overset{T}{\int }}{\tau }_{W}dt|}{\underset{0}{\overset{T}{\int }}|{\tau }_{W}|dt}\right)$ $\text{TAWSS}=\frac{1}{T}\underset{0}{\overset{T}{\int }}|{\tau }_{W}|dt$	Thrombotic susceptibility due to the activation/dysfunction of endothelial cells

*WSS* wall shear stress, *ROI* region of interest, $\mu$ dynamic viscosity, y distance from the wall, *ECAP* endothelial cell activation potential, *OSI* oscillation index, *T* integration period, *TAWSS* time-averaged wall shear stress

### Data acquisition and 3D reconstruction

Cardiac computed tomography (CT) images of a patient, who underwent LAAO with a 27 mm Watchman Gen 2.5 device, were used in this in silico study. Thoracic CT images were acquired with a 320-detector scanner Aquilion One (Toshiba Corp., Minato, Tokyo, Japan) using 50 ml contrast agent Visipaque 320 injection, scans were made at mid-systole and mid-diastole. Pre- and post-operative CT images were available. In this study, pre-operative images at mid-systole were used to reconstruct the anatomic model for virtual device implantation, whereas post-operative images at mid-systole were only analyzed to determine the clinically implanted position of the LAAO device. Written informed consent from the patient was obtained prior to processing of the data.

The CT dataset was processed in the open-source software 3D Slicer. The in-plane pixel size was 0.5 × 0.5 mm^2^ and the slice gap was 0.5 mm, resulting in a 3D image matrix of 0.5 × 0.5 × 0.5 mm^3^. In both the pre- and post-operative datasets, the reconstructed image matrix size was 512 × 512 × 640. Semi-automatic techniques, including thresholding, region growing and manual corrections, were used to create a binary mask that marks the left atrium (LA) including the LAA structure in the CT images. Based on the LA/LAA binary mask, 3D LA/LAA anatomic structure was constructed using the Marching Cubes method. The anatomic model was thereafter smoothed and re-meshed using the software MeshMixer (Autodesk, Inc. San Rafael, CA, USA). Pulmonary veins (PVs) beyond the first bifurcation were removed. For the FEM virtual device implantation in the next step, the reconstructed LA/LAA model was given a wall thickness of 1 mm.

### Modeling and virtual implantation of LAAO device

For the simulation of the non-pacifier device, the Gen 2.5 Watchman 27 was used for ostial positions and Watchman 24 was used for deep implantation, as this position would only be achievable with a smaller device in the given anatomy. The Watchman 27 and Watchman 24 occluders were assumed to have a diameter of 24 mm and 21.6 mm after their deployment in the LAA (11% and 10% compression). These diameters were defined so that the expanded device fitted their target landing zones with compression rates that fulfilled the releasing criteria of 8–20% [23]. The occluder’s skirt with the morphology after deployment was modeled in Inventor (Autodesk, Inc. San Rafael, CA, USA). Thereafter, virtual device implantation was performed using FEM solver ABAQUS explicit (Dassault Systèmes, Vélizy-Villacoublay, France). The LA/LAA tissue was modeled as hyper-elastic material using the Mooney-Rivlin strain energy function, with the parameters from a previously developed LA model [24]. In the FEM simulation, the LA model was meshed into 237,402 tetrahedral elements and used for the simulations of both non-pacifier and pacifier occluders. The Watchman device was split into ten segments in the FEM model, each segment was defined as a rigid surface and meshed with 1309 quad-dominated elements. For the virtual device deployment, the LAAO device was firstly collapsed, in that each of the ten segments moved 7 mm inwards along the radial direction. After being transported into the target position, the device was expanded, in that each segment moved back to its original position. LA/LAA tissue deformation occurred during this process.

For pacifier devices, the nitinol disc structure of the Amplatzer Amulet LAA occluder was modeled in the CAD software Inventor (Autodesk, Inc. San Rafael, CA, USA) and meshed in FEM solver ABAQUS explicit (Dassault Systèmes, Vélizy-Villacoublay, France) into 12,000 beam elements. The nitinol material properties were taken from the literature [25]. For the virtual implantation, the disc of the Amulet occluder was firstly placed proximally to the LAA ostium, the displacement into the LAA was subsequently defined to the disc’s middle (threaded insert), so that the disc moved to its target position while keeping LA/LAA tissue deformation into account. Amulet 25 (disc diameter 32 mm) and 22 (disc diameter 28 mm) were virtually implanted, as the landing zone width of the selected patient’s LAA fitted for both Amulet sizes according to the instruction provided by the device manufacturer. The lobe was manually reconstructed in the software MeshMixer (Autodesk, Inc. San Rafael, CA, USA) for visualization. This component, however, has no effect on the hemodynamics in the LA.

This study contains eight simulated LAAO scenarios and one pre-LAAO model. Four positions of the non-pacifier (NP) occluder were simulated: (i) clinically implanted device position according to post-operative CT data (NP-CL position); (ii) ostium-fitted position (NP-OS position); (iii) position with severe device tilt towards the mitral valve (MV; NP-TL position) and (iv) deep implantation with an axial device offset to the distal LAA apex (NP-DS position). Similarly, ostium-fitted and distal positions were replicated with both the larger pacifier occluder (LP-OS, LP-DS positions) and the smaller pacifier device (SP-OS, SP-DS positions). A severely tilted position towards the MV (TL) is not feasible for the pacifier device in the given anatomy. After virtual implantation, device positions were characterized in the cut plane that simulated the lower-middle transesophageal view with 90° rotation, which is clinically used to determine the position of the LAAO device. Left upper pulmonary vein (LUPV) ridge length, implant depth towards the MV and angle between the device and the LUPV ridge were measured.

### Computational fluid dynamics

**Fig. 1:**
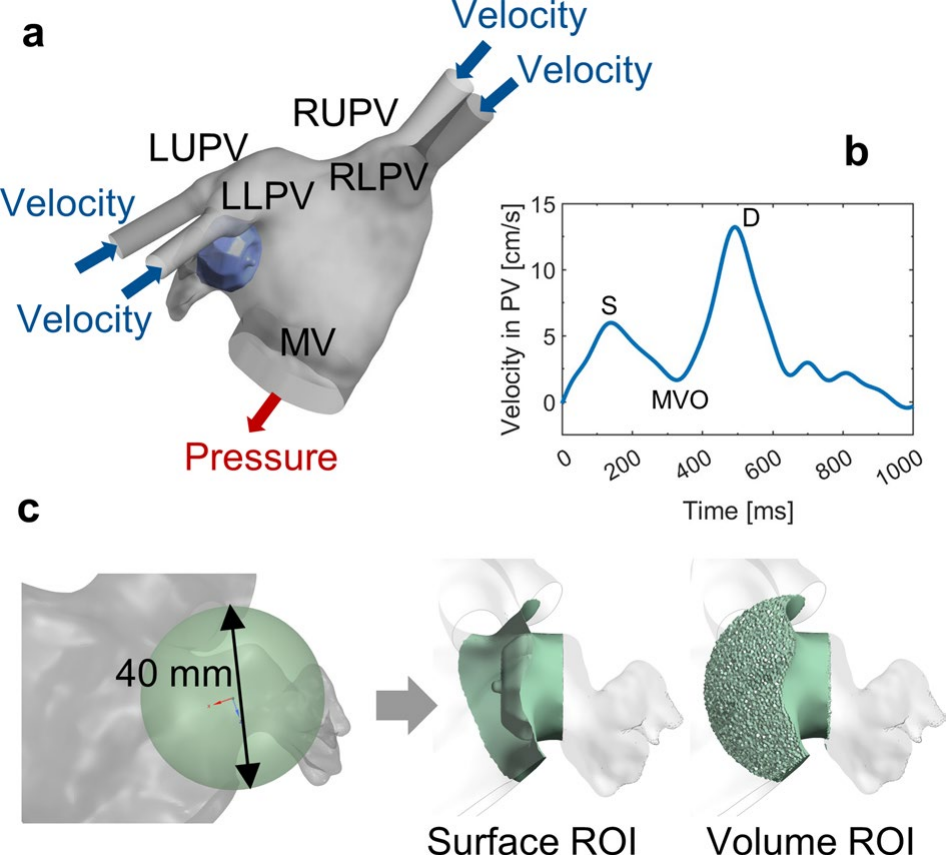
**a** Boundary conditions for the CFD model: PVs as velocity-inlet (blue) and MV as pressure-outlet (red); **b** Inflow velocity at each PV is defined according to the flow pattern in AF; **c** Definition of ROI. For all the device positions and the pre-LAAO model, ROI is uniformly defined as the region of the LA contained within a sphere with a diameter of 40 mm, centered on the middle point of the LAA ostium. In LAAO models, a distal end of the LAAO device was excluded from the ROI. *LAAO* left atrial appendage occlusion, *LA* left atrium, *LUPV* left upper pulmonary vein, *LLPV* left lower pulmonary vein, *RUPV* right upper pulmonary vein, *RLPV* right lower pulmonary vein, *LAA* left atrial appendage, *MV* mitral valve, *MVO* mitral valve opening, *ROI* region of interest

The hemodynamics of the eight LAAO scenarios, as well as in the native LA/LAA anatomy, were assessed through CFD simulations in a dynamic regime. Simulations were performed in ANSYS Fluent (ANSYS Int., Canonsburg, PA, USA). Following the FEM device implantation, the LA/LAA internal volume was extracted and used as the fluid domain in the CFD simulation. Four 30 mm inlet tubes and one 10 mm outlet tube were added onto the PVs and the MV, respectively (Fig. 1a). Volumetric grid with polyhedral mesh elements was generated for all the models and the final meshes counted approx. 500,000 in each model.

Blood was assumed as Newtonian, incompressible fluid with a density of 1060 kg/m^3^ and dynamic viscosity of 0.0035 Pa·s. The maximum Reynolds number (Re) was approx. 1900 (Re < 2300) and, therefore, a laminar flow model was applied. To simulate the AF hemodynamic condition, transient velocity inflow was assigned to the four PVs according to the clinical measurement [26, 27]. The MV was defined as pressure outflow with zero gauge pressure, as displayed in Fig. 1a and b. Surfaces of the LA anatomy and the LAAO device were set as rigid walls with no-slip condition. The volume in front of the device’s atrial surface was considered as region of interest (ROI) to assess DRT. The ROI for all models was defined as the region of the LA contained within a sphere with a diameter of 40 mm centered on the middle point of the LAA ostium, excluding the region of the appendage distal to the LAAO device, as displayed in Fig. 1c.

When solving each CFD model, ten initial cardiac cycles were run to avoid the influence of the non-physiological initial condition on fluid velocity. Six further cardiac cycles were simulated and used to evaluate hemodynamics. The total time domain was discretized with a fixed time step of 0.005 s.

### Evaluation of blood flow pattern and residual blood

Streamlines of blood flow in the LA/LAA for different device positions were visualized at the beginning of mitral valve opening (MVO) and in mid-diastole (D-wave), as these two-time points represent the lowest and highest inlet flow rate at the pulmonary veins, respectively. The volume-averaged velocity and vorticity were calculated in the ROI during the last cardiac cycle.

The residual blood was visualized after the simulated six cardiac cycles. For quantitative evaluation, the volume fraction of residual blood with respect to the first cycle within the ROI (*φ* = *V*_old blood_/*V*_ROI_) and the washout half-time *t*_*h* ( φ= 50%)_ were calculated for each device position.

### Evaluation of wall shear stress

Time-averaged WSS (TAWSS) was determined for the LA/LAA and occluder surfaces included in the ROI to visualize the potential areas of platelet adhesion. The mean TAWSS was calculated during the last cardiac cycle. In the LA, transient discoid platelet aggregates could be potentially formed at the pulmonary vein limbus due to the high local wall shear stress and endothelial cell activation potential. When these platelet aggregates are exposed to low shear stress at downstream (e.g. in the “cul-de-sac”), they physically restructure, increasing the strength and stability of discoid platelet aggregates, thereby promoting thrombus growth [28]. Previous work in artery wall regions indicated that monocyte/cell adhesion is expected to occur if wall shear stress falls below 0.36 Pa [29–32].

### Evaluation of endothelial cell activation potential

The ECAP index represents the thrombotic susceptibility due to the activation of endothelial cells and is defined as the ratio between the oscillation index (OSI) and TAWSS [33]

1 $$\text{ECAP}=\frac{\rm{OSI}}{\rm{TAWSS}},$$

OSI is a non-dimensional index that indicates the complex and irregular changes in blood flow patterns that are related to blood coagulation and is calculated as

2 $$\text{OSI}=\frac{1}{2}\left(1-\frac{|\underset{0}{\overset{T}{\int }}{\tau }_{W}dt|}{\underset{0}{\overset{T}{\int }}|{\tau }_{W}|dt}\right),$$

where τ_W_ is the instantaneous WSS vector and T is the integration period. TAWSS is calculated as

3 $$\text{TAWSS}=\frac{1}{T}\underset{0}{\overset{T}{\int }}|{\tau }_{W}|dt.$$

Kelsey et al. reported critical thrombotic susceptibility in the areas where endothelial cell activation potential is above 1.4 Pa^−1^ [34]. In this study, ECAP on the LA/LAA and occluder surfaces included in the ROI was determined and the area-averaged ECAP was calculated.

## Results

### Device positions after virtual implantation

**Fig. 2:**
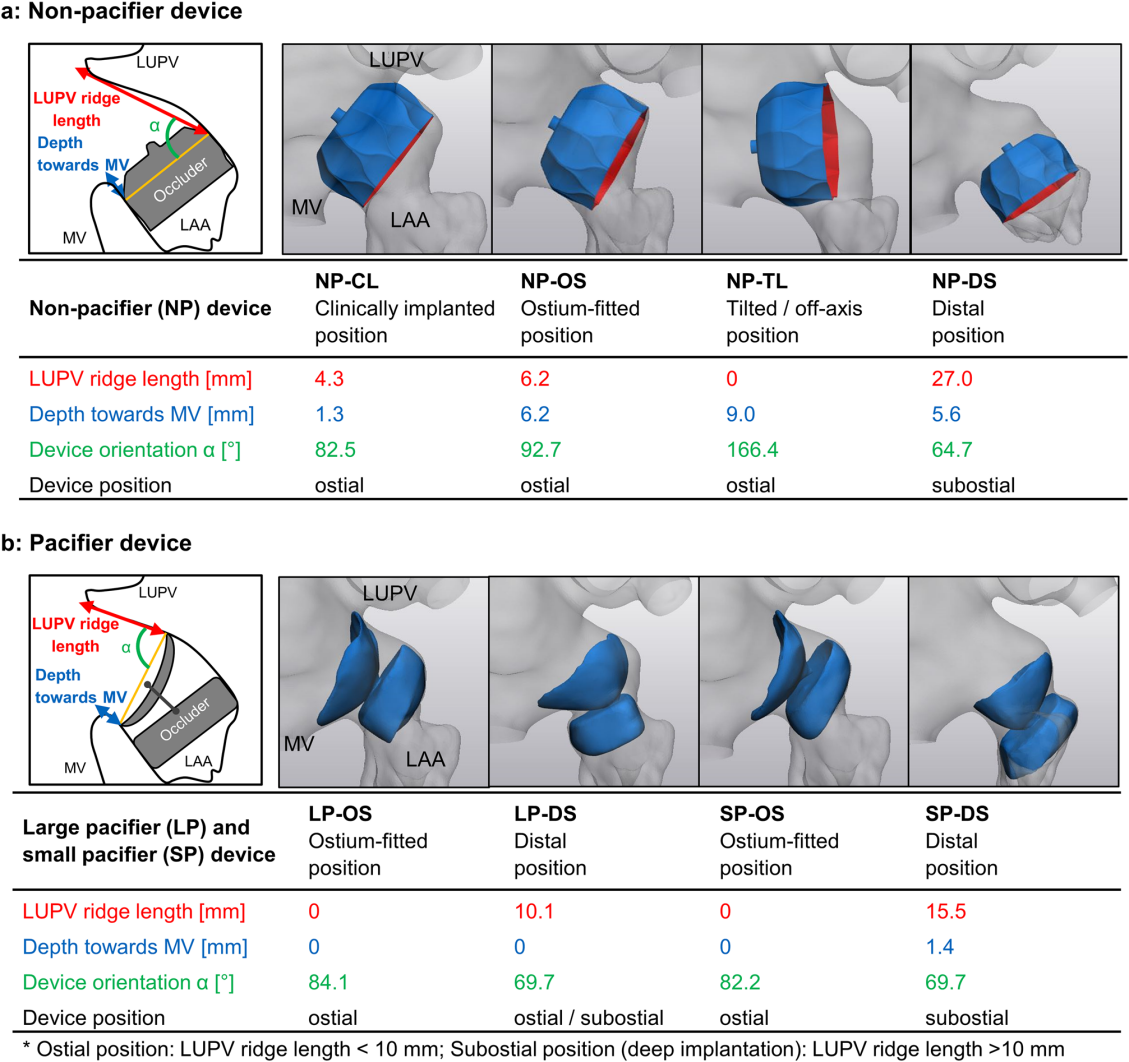
**a** Virtually implanted non-pacifier LAAO device with different positions and results of the device position measurement. **b** Virtually implanted pacifier LAAO device with different positions and results of the device position measurement. *LA* left atrium, *LAA* left atrial appendage, *LAAO* left atrial appendage occlusion, *LUPV* left upper pulmonary vein, *MV* mitral valve

Non-pacifier and pacifier LAAO devices were virtually implanted to generate eight different scenarios. For the non-pacifier device, the NP-DS position represented a deep implantation (defined as LUPV ridge length > 10 mm [35]) that left a “cul-de-sac” from the pulmonary vein limbus to the LAAO device (LUPV ridge length: 27 mm). The NP-TL position was off-axis (*α* = 166.4°) and resulted in a cavity towards the MV, while the clinically implanted non-pacifier device (NP-CL) was positioned slightly proximal compared to the ostial position (NP-OS; Fig. 2a). For the pacifier devices, the distal positions resulted in an uncovered part of the LUPV ridge, however, with different lengths. The SP-DS position represented deep implantation (LUPV ridge length: 15.5 mm) with a “cul-de-sac” in the LAA (Fig. 2b), while the LUPV ridge length of LP-DS position (10.1 mm) was on the border between deep implantation and ostial position [35] and the resulting ‘cul-de-sac’ was smaller.

### Blood flow pattern

**Fig. 3:**
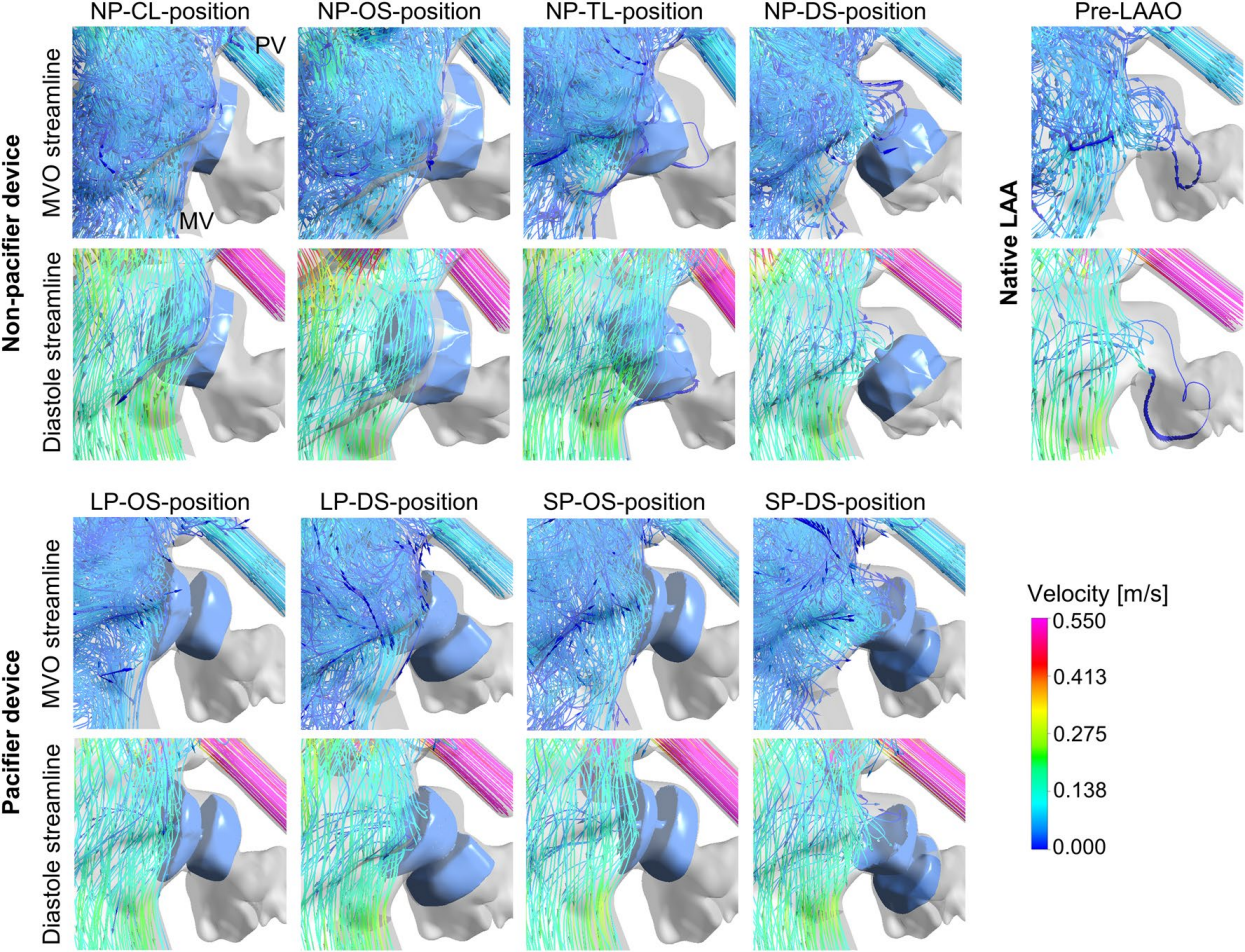
Streamlines of blood flow prior and post to LAAO with different device types and positions; *LAAO* left atrial appendage occlusion, *ROI* region of interest, *MVO* mitral valve opening, *PV* pulmonary vein, *MV* mitral valve

**Fig. 4:**
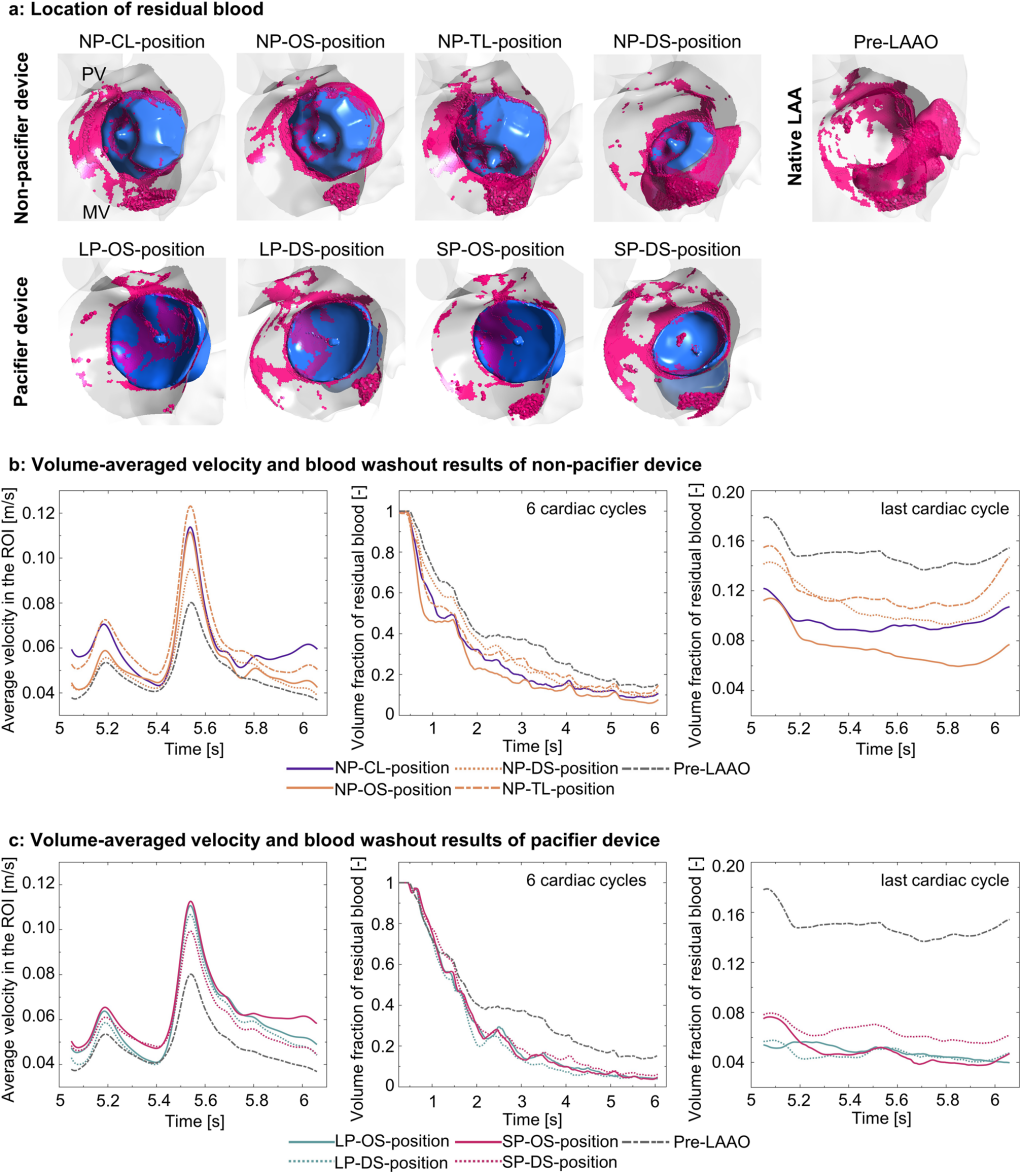
**a** Residual blood in the ROI prior and post to LAAO with different device types and positions; **b** Volume-averaged velocity in the ROI, volume fraction of residual blood in the ROI prior and post to LAAO with the non-pacifier device and **c** with the pacifier device. *LAAO* left atrial appendage occlusion, *ROI* region of interest, *PV* pulmonary vein, *MV* mitral valve

Table 2 displays a summary of the hemodynamic results. In the pre-LAAO model, blood entered the LAA with a low velocity (0.041 m/s at mitral valve opening and 0.065 m/s at mid-diastole). In contrast, the presence of the LAAO with both types of device prevented blood flow into the LAA, except for the tilted device position of the non-pacifier device (NP-TL), where streamlines went into the LAA (Fig. 3a). The volume-averaged velocity in the ROI volume during the last cardiac cycle is displayed in Fig. 4b and c, with the pre-LAAO model presenting the lowest average velocity. Overall, deep implantation resulted in a lower average blood velocity compared to the ostial position. The velocities within the ROI were higher with the pacifier device than with the non-pacifier device.

**Table 2:** Summary of hemodynamic results

	Pre-LAAO	Non-pacifier occluder				Pacifier occluder			
		NP-CL	NP-OS	NP-TL	NP-DS	LP-OS	LP-DS	SP-OS	SP-DS
Hemodynamic characteristics									
Average velocity [m/s]	0.048	0.061	0.054	0.063	0.053	0.065	0.066	0.066	0.061
Average vorticity [m^3^/s]	2.81·10^–4^	2.53·10^–4^	2.71·10^–4^	2.71·10^–4^	3.62·10^–4^	3.26·10^–4^	3.52·10^–4^	3.57·10^–4^	4.04·10^–4^
Washout half-time [s]	1.73	1.11	0.81	1.43	1.63	1.49	1.48	1.53	1.60
Volume fraction of residual blood after six cardiac cycles [–]	0.15	0.11	0.08	0.15	0.12	0.04	0.05	0.05	0.06
Average WSS [Pa]	0.22	0.28	0.26	0.26	0.21	0.32	0.31	0.35	0.30
Average ECAP [Pa^−1^]	0.45	0.25	0.85	1.10	2.23	0.147	0.154	0.096	0.157

*LAAO* left atrial appendage occlusion, *WSS* wall shear stress, *ECAP* endothelial cell activation potential

### Residual blood

In all the LAAO models, residual blood was found in the volume between the device’s lateral surface and the LUPV ridge/MV annulus, as well as around the threaded insert on the devices’ atrial face (Fig. 4a, Supplementary Animations 1 & 2). Among all the simulated scenarios, the pre-LAAO model and the off-axis position of the non-pacifier device presented the most residual blood after six cardiac cycles (15%).

For the Watchman device, the ostium-fitted (NP-OS) position exhibited the most efficient blood washout with the shortest washout half-time, whereas the distal position (NP-DS) and the pre-LAAO models had the longest washout half-time (NP-DS: 1.63 s, pre-LAAO: 1.73 s vs NP-OS: 0.81 s). Compared to the ostium-fitted position, significantly more residual blood in the ROI was found in the clinical (NP-CL), off-axis (NP-TL) and distal (NP-DS) positions (NP-CL: 11%, NP-TL: 15% and NP-DS: 12% vs NP-OS: 8%; Fig. 4b).

The pacifier device generally led to longer washout half-time than the non-pacifier device. However, less residual blood after six cardiac cycles was associated with the pacifier device. Deep implantation of the smaller pacifier device resulted in slightly more residual blood in comparison to the ostial position (SP-DS: 6% vs SP-OS: 5%), while no difference was found among the positions of the larger device (Fig. 4c).

### Wall shear stress

**Fig. 5:**
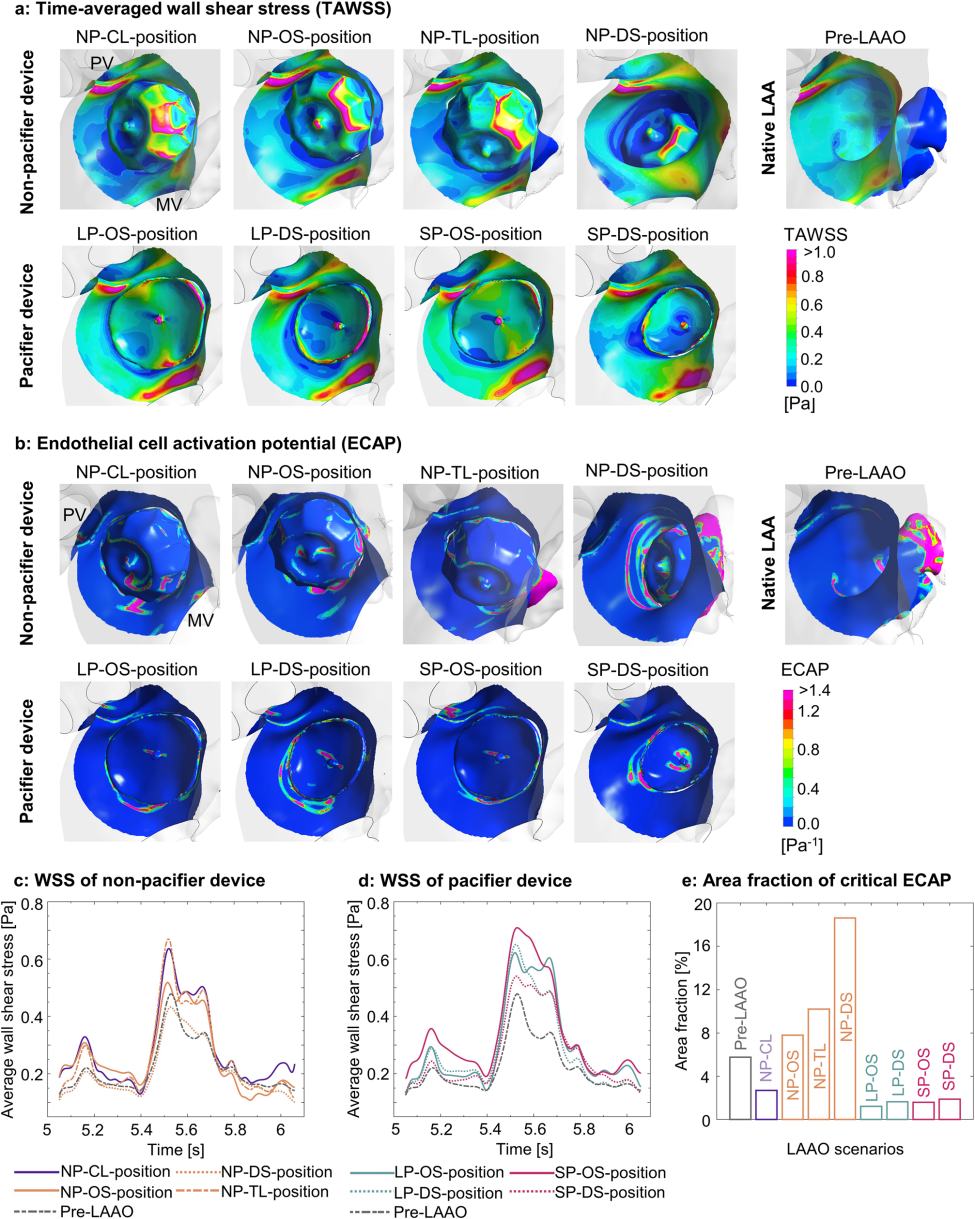
**a** Time-averaged wall shear stress (TAWSS) on the ROI surface prior and post to LAAO with different device types and positions; **b** Endothelial cell activation potential (ECAP) on the ROI surface prior and post to LAAO with different device types and positions; **c** Area-averaged wall shear stress of non-pacifier device; **d** Area-averaged wall shear stress of pacifier device; **e** The area fraction of critically high ECAP in the ROI. *LAAO* left atrial appendage occlusion, *ROI* region of interest, *PV* pulmonary vein, *MV* mitral valve

In the simulated LAAO scenarios, the location of areas of critically low TAWSS (< 0.36 Pa [29–32]) corresponded to the regions with residual blood accumulation, i.e. the atrial surface surrounding the threaded insert, as well as the devices’ lateral surface and the neighboring tissue (Fig. 5a). Regarding the effect of the device position, deep implantation led to lower TAWSS with the non-pacifier (NP-DS: 0.21 Pa vs NP-OS: 0.26 Pa) and the small pacifier device (SP-DS: 0.30 Pa vs SP-OS: 0.35 Pa), while no difference was found among the positions of the large pacifier device. In addition, all ostial device positions resulted in higher average WSS in comparison to pre-LAAO (0.22 Pa; Fig. 5c and d). The pacifier device generally resulted in a higher time-averaged WSS in the ROI.

### Endothelial cell activation potential

The areas of critically high ECAP (> 1.4 Pa^−1^ [34]) were mainly distributed on the devices’ atrial surface surrounding the threaded insert, as well as on the device’s lateral surface and the neighboring tissue (Fig. 5b). Compared to the ostial position, deep implantation with the non-pacifier and the smaller pacifier device was associated with increased average ECAP due to the “cul-de-sac” (NP-DS: 2.23 Pa^−1^ vs NP-OS: 0.85 Pa^−1^; SP-DS: 0.16 Pa^−1^ vs SP-OS: 0.10 Pa^−1^). This effect was, however, not appreciable with the larger pacifier device. Of note, lower average ECAP values were observed with the pacifier device, compared to the non-pacifier device (Table 2). Deep implantation of the non-pacifier device resulted in a larger area fraction of critically high ECAP in the ROI (NP-DS: 18.6% vs NP-OS: 7.8%), while for the pacifier device the area fraction of high ECAP is generally smaller and the area fraction increased only slightly with deep implantation (SP-DS: 1.9% vs SP-OS: 1.6%; LP-DS: 1.7% vs LP-OS: 1.2%; Fig. 5e).

## Discussion

In recent years, the potentially detrimental impact of DRT on outcomes after LAAO has been highlighted [5–7]. Although an ostium-fitted device position with complete sealing is desirable, the complexity and variations of the human LAA, as well as procedure-related factors make deployment of the LAAO device in an optimal position not always achievable [36]. During device release, deep implantation in a distal position can occur even if release criteria are fulfilled [23]. As implant- and procedure-specific aspects appear to play a role, there is a need for a better understanding of DRT formation and prevention. Unfortunately, the limited ability to assess local flow conditions surrounding LAAO devices renders clinical assessments of these effects practically impossible. As a potential solution to this issue, in silico models represent a promising tool to provide mechanistic insights into clinical observations.

The present study made use of in silico modeling by including the precise morphology of non-pacifier and pacifier LAAO devices, as well as their interaction with LAA tissue, to evaluate the impact of device position on local hemodynamics. In this context, potential markers for thrombogenicity, including blood stasis, platelet adhesion and endothelial dysfunction, were determined and studied. We looked at three markers to have a more precise prediction of thrombosis as suggested by an in silico study for intracranial aneurysms, where the combination of wall shear stress with blood residence time was shown to be a better predictor than wall shear stress alone [37]. As a result, we were able to elucidate the role of implant type as well as position on surrogate markers associated with thrombosis.

As for the non-pacifier device, the clinical and ostium-fitted positions resulted in low markers of DRT. In comparison, deep implantation (LUPV ridge length: 27 mm; NP-DS) showed increased potential DRT specifically in terms of (1) decreased blood washout around the device; (2) increased endothelial cell activation potential and (3) lower average wall shear stress. In fact, local hemodynamics in the “cul-de-sac” resembled stasis similar to the native LAA and thus a prothrombotic environment. These results are in line with recent clinical observations. For instance, Kaneko et al. [38] reported that the device was implanted in a deep position in 75% (3/4) of DRT patients treated with Watchman. For the pacifier device, deep implantation (LUPV ridge length: 15.5 mm; SP-DS) was also associated with higher potential DRT risk with respect to (1) the effect of “cul-de-sac” on blood stasis; (2) endothelial cell dysfunction and (3) platelet aggregate adhesion. This finding is also coherent with previous clinical observations. In fact, the studies by Freixa et al. [8] and Aminian et al. [39] found that in patients treated with a pacifier device, an uncovered LUPV ridge resulted in a higher incidence of DRT and DRT were mostly observed in the “cul-de-sac” [40]. In the clinical observational cohorts with both types of devices, Simard et al. [10] identified deep implantation as an independent risk factor for DRT and patients with DRT were noted to have a larger average implantation depth [7, 9, 11, 35].

With both device types, the deep implantation was achieved with a smaller device size, which pointed to the danger of undersizing the device. Complete coverage of the LUPV ridge is not always achievable due to the anatomy and access to the LAA. In this case, a smaller device size has a higher risk of deep implantation. In clinical practice, device undersizing was commonly observed in patients with DRT [39].

In addition to deep implantation, the off-axis position of the non-pacifier device (NP-TL) also exhibited a higher potential DRT risk (i.e. worse surrogate markers of stasis and endothelial dysfunction) compared to the optimal positions. In this position, a cavity between the LAAO device and the mitral valve was formed, which could act as a blood stagnation zone. In fact, residual blood accumulation and areas of critically high ECAP were observed in this cavity.

Our study suggests that device-specific aspects play a role in DRT: the pacifier device exhibited a lower potential DRT risk compared to the non-pacifier device. Due to its shape after implantation, fewer critical areas for low flow were observed on the pacifier device: Its disc has a small lateral surface and often a more proximal contact zone to the LAA wall with less critical hemodynamic conditions. In contrast, the shoulder of a non-pacifier device and the neighboring tissue create a large thrombus-prone region associated with higher markers of DRT (Fig. 4a**, **Fig. 5a and b**)**, these specific areas have been identified as predisposing nidus for DRT formation in clinical studies [20, 22]. Because of the small critical area for low flow, the pacifier device implanted with a depth of 10.1 mm (LP-DS) did not show increased blood stasis compared to the ostial position.

Of note, several clinical studies reported a lower DRT prevalence with the pacifier device than with the non-pacifier device [41–43], which is in line with our findings. Hereby it remains to be said that it is impossible for clinical studies to compare the hemodynamics in patients under the same condition by eliminating other factors, e.g. AF status, history of stroke, decreased ejection fraction, periprocedural management, postprocedural discharge on anticoagulants [5, 6] and microinjury caused by the device. These non-hemodynamic factors could also explain why the DRT prevalence is varying significantly between the different clinical observations, including reports that the non-pacifier device leads to lower DRT prevalence than the pacifier device [7, 11].

Besides the lateral surface of the device and the neighboring tissue, another critical area for DRT was the cove around the threaded insert. In our study, residual blood, risk of platelet adhesion assessed as wall shear stress (i.e. WSS < 0.36 Pa [29–32]), and endothelial cell dysfunction (i.e. ECAP > 1.4 Pa^−1^ [34]) were also found in these areas with both non-pacifier and pacifier devices, thus identifying them as regions of high DRT risk. In fact, in an analysis from our group [7], 58.6% and 50% DRT were detected around the threaded insert on non-pacifier and pacifier devices, respectively.

We believe our study was able to reproduce the clinical observations because of the precise geometry of the device model, in contrast to other studies [14, 15, 19] presenting simplified geometry and failing to identify those areas of high DRT.

In summary, our proposed in silico model provides a potential pathophysiological and mechanistic basis for the impact of device position and local hemodynamics on DRT formation after LAAO and highlights the need for optimal device position, confirming clinical observation. To achieve an optimal device position in clinical practice, preprocedural planning and periprocedural guidance for device selection are crucial. In this context, the use of preprocedural computational modeling based on cardiac CT, rapid prototyping, as well as optimized device conformability and delivery sheaths could prove to be beneficial.

## Limitation

This study has several limitations: as specified in the methods, in the CFD simulations the boundary condition for the pulmonary vein inflow was taken from published echocardiographic recordings of a patient with atrial fibrillation [26], as such measurements were not included in the clinical data available for this study. While the complexity of LAA anatomy is vast, only one specific anatomical model was used. With the given anatomy, streamlines entering the LAA were observed with the off-axis position of a non-pacifier device. Further simulations with various anatomies are needed to verify the capability of our in silico model in replicating peri-device leak occurrence. Non-hemodynamic factors that may be crucially important for DRT, including endothelial microinjury caused by the device, AF status, history of stroke, decreased ejection fraction, periprocedural management and postprocedural medication [5, 6] cannot be assessed with the CFD model.

## Conclusion

In this study, we assessed the influence of the LAAO device type and position on the potential risk of DRT using in silico model that included the precise geometry of the LAAO device and its interaction with the LA tissue during virtual implantation. The results revealed that a deep implantation as well as an off-axis device position were associated with increased potential DRT risk and the pacifier device showed lower potential risk for DRT compared to the non-pacifier device, as assessed in terms of blood stasis, platelet adhesion and endothelial dysfunction. These findings provide a mechanistic basis for recent clinical observations and emphasize the importance of obtaining an optimal device position when performing an LAAO procedure.

## Supplementary Information

Below is the link to the electronic supplementary material.

**Animation 1:** Visualization of residual blood (red) in the LA with different non-pacifier LAAO device positions in 6 cardiac cycles (t = 6.06 s). LAAO: left atrial appendage occlusion. (MP4 11446 KB)

**Animation 2:** Visualization of residual blood (red) in the LA with different pacifier LAAO device positions in 6 cardiac cycles (t = 6.06 s). LAAO: left atrial appendage occlusion (MP4 11577 KB)

## Data Availability

The datasets generated and analysed during the current study are available from the author on reasonable request.

## Abbreviations and acronyms

AF: Atrial fibrillation
CFD: Computational fluid dynamics
CT: Computed tomography
DOACs: Direct oral anticoagulants
DRT: Device-related thrombus
ECAP: Endothelial cell activation potential
FEM: Finite element method
LA: Left atrium
LAA: Left atrial appendage
LAAO: Left atrial appendage occlusion
LUPV: Left upper pulmonary vein
MV: Mitral valve
MVO: Mitral valve opening
OSI: Oscillation index
PDL: Peri-device leak
PV: Pulmonary vein
Re: Reynolds number
ROI: Region of interest
TAWSS: Time-averaged wall shear stress
WSS: Wall shear stress
